# Supplementary material for: MetaRibo-Seq measures translation in microbiomes
Source: Nat Commun. 2020 Jun 29;11:3268. doi: 10.1038/s41467-020-17081-z (PMC7324362; doi:10.1038/s41467-020-17081-z)
Supplement: Supplementary file 10 — Supplementary Data 7 [file 41467_2020_17081_MOESM10_ESM.zip › File2/Confidence_High_Taxonomy/207575_out.krona.html]

Javascript must be enabled to view this page.

members
magnitude
magnitudeUnassigned
count
unassigned
taxon
rank

207575\_out

107

107
2
superkingdom

107
phylum
1239

186801
class
107

order
186802
107

family
216572
1

genus
459786
1

1

SRS101433\_contig\_number\_4217
1897011
species

family
541000
106

2
genus
204475

2

SRS1041129\_contig\_number\_10278SRS143598\_contig\_number\_22564
745368
species

216851
genus
32

32
1946508

SRS022137\_contig\_number\_7553SRS024331\_contig\_number\_13529SRS046369\_contig\_number\_10156SRS047014\_contig\_number\_contig-100\_7339.113439SRS049402\_contig\_number\_4324SRS050422\_contig\_number\_contig-100\_5702.77306SRS050925\_contig\_number\_27375SRS051882\_contig\_number\_contig-100\_13541.85050SRS055982\_contig\_number\_7010SRS057480\_contig\_number\_6478SRS058145\_contig\_number\_contig-100\_14868.14869SRS063518\_contig\_number\_contig-100\_4695.186191SRS065176\_contig\_number\_contig-100\_26977.26978SRS075773\_contig\_number\_contig-100\_20472.20473SRS078242\_contig\_number\_4712SRS098514\_contig\_number\_contig-100\_3068.3068SRS1041136\_contig\_number\_contig-100\_1030.76874SRS104485\_contig\_number\_contig-100\_6375.6375SRS1055069\_contig\_number\_contig-100\_1060.38251SRS1055076\_contig\_number\_contig-100\_2105.2106SRS140492\_contig\_number\_contig-100\_5021.163434SRS142503\_contig\_number\_23445SRS142599\_contig\_number\_18714SRS144537\_contig\_number\_17504SRS144714\_contig\_number\_26067SRS145497\_contig\_number\_contig-100\_4499.4499SRS146813\_contig\_number\_contig-100\_5164.112820SRS147425\_contig\_number\_contig-100\_1057.1058SRS147614\_contig\_number\_contig-100\_2059.2060SRS148159\_contig\_number\_contig-100\_20009.233161SRS148721\_contig\_number\_36257SRS971276\_contig\_number\_8851
species

68
species

SRS042628\_contig\_number\_contig-100\_13606.119603SRS042966\_contig\_number\_8400SRS045826\_contig\_number\_8678SRS050752\_contig\_number\_23446SRS050998\_contig\_number\_contig-100\_68.50286SRS051031\_contig\_number\_contig-100\_323.87582SRS051610\_contig\_number\_2731SRS052697\_contig\_number\_14444SRS053214\_contig\_number\_20862SRS054956\_contig\_number\_8346SRS054956\_contig\_number\_contig-100\_11645.11645SRS056273\_contig\_number\_20468SRS058770\_contig\_number\_18806SRS063040\_contig\_number\_28441SRS063985\_contig\_number\_25566SRS064276\_contig\_number\_34760SRS075078\_contig\_number\_28949SRS075878\_contig\_number\_15722SRS075984\_contig\_number\_26036SRS076756\_contig\_number\_7323SRS077127\_contig\_number\_10788SRS077194\_contig\_number\_29077SRS077231\_contig\_number\_4465SRS077392\_contig\_number\_40044SRS097920\_contig\_number\_15225SRS098571\_contig\_number\_44658SRS098655\_contig\_number\_12417SRS098881\_contig\_number\_26583SRS104084\_contig\_number\_8875SRS1041033\_contig\_number\_contig-100\_9257.135936SRS1041136\_contig\_number\_8824SRS104327\_contig\_number\_3611SRS104400\_contig\_number\_contig-100\_28656.64872SRS104636\_contig\_number\_25605SRS104912\_contig\_number\_16815SRS105153\_contig\_number\_25961SRS1055056\_contig\_number\_8426SRS1055067\_contig\_number\_13848SRS142542\_contig\_number\_25008SRS142890\_contig\_number\_37683SRS142923\_contig\_number\_contig-100\_15426.64877SRS143148\_contig\_number\_2740SRS143417\_contig\_number\_1197SRS143466\_contig\_number\_18065SRS143895\_contig\_number\_31485SRS143991\_contig\_number\_26596SRS144135\_contig\_number\_24219SRS144183\_contig\_number\_37184SRS144297\_contig\_number\_11818SRS144362\_contig\_number\_42464SRS144753\_contig\_number\_9432SRS146812\_contig\_number\_contig-100\_2806.256420SRS147346\_contig\_number\_42095SRS147377\_contig\_number\_5137SRS147653\_contig\_number\_2505SRS147919\_contig\_number\_13197SRS147977\_contig\_number\_12163SRS148196\_contig\_number\_34382SRS148253\_contig\_number\_12285SRS148319\_contig\_number\_contig-100\_9598.9599SRS148511\_contig\_number\_17062SRS148784\_contig\_number\_22497SRS148817\_contig\_number\_9433SRS149784\_contig\_number\_contig-100\_2596.154450SRS149879\_contig\_number\_24879SRS893172\_contig\_number\_13711SRS893279\_contig\_number\_15254SRS893373\_contig\_number\_4352
1898205

292632
genus
4


SRS022524\_contig\_number\_9024SRS024009\_contig\_number\_11835SRS043411\_contig\_number\_15900SRS147557\_contig\_number\_26045
1897022
species
4
